# Retained intra-abdominal surgical sponge causing ileocolic fistula diagnosed by colonoscopy

**DOI:** 10.1016/j.ijscr.2020.01.018

**Published:** 2020-01-23

**Authors:** Asaad Shareef Omar, Ayad Ahmad Mohammed

**Affiliations:** Department of Surgery, College of Medicine, University of Duhok, Kurdistan Region, Iraq

**Keywords:** Retained surgical sponges, Textiloma, Gossypiboma, Colonoscopy, Ileocolic fistula

## Abstract

**Introduction:**

Retained surgical sponges and instruments is a well-recognized medical error that may occur after all kinds of surgeries. This event has a catastrophic impact on the patient, health care workers, and the health institution. Sometimes, it is termed as textiloma or gossypiboma.

**Case presentation:**

A 40-year-old lady presented with abdominal pain, diarrhea and bilious vomiting for 3 days. The patients had history of cesarean section which was performed before 4 months. During examination she was pale and she had tenderness in the lower abdomen. CT-scan of the abdomen showed thickening of the wall of the sigmoid colon with evidence of intramural air and dilated small bowel loops. Colonoscopy showed evidence of surgical sponge causing transmural erosion and ulceration of the sigmoid colon. During surgery there was an evidence of a retained surgical sponge resulting in fistula between the ileum and the sigmoid colon. Resection of the involved part of the ileum and the sigmoid colon was done with end-end anastomosis. After 10 days she developed complete abdominal dehiscence. An emergency operation was performed for the patient and the abdomen was closed with tension sutures.

**Conclusion:**

The surgical team is responsible for preventing this event by careful inspection of the surgical site using all the available methods and technology. Technology increases the safety but doesn’t accurately prevent the accidents. All causative human and technical factors must be addressed carefully.

## Highlights

• Retained surgical sponges and instruments is a well-recognized medical error.
• Some medical errors are potentially preventable.
• The surgical team is responsible for preventing such events.

## 1 Introduction

Retained surgical sponges and instruments is a well-recognized medical error that may occur after all kinds of surgeries. This event has a catastrophic impact on the patient, health care workers, and the health institution. This event is listed as one of the 27 ‘never events’ which is released by the National Quality Forum in the United States and is also included in the guidance which is issued by the UK Department of Health [1,2].

Sometimes retained surgical sponge is termed textiloma (which is derived from the Latin word “textile” and oma, meaning “swelling”), or gossypiboma (which is derived from the Latin word Gossypium, the genus of cotton plants [3].

Some of these errors are potentially preventable like operation for the wrong patient, wrong site, wrong side, and the retention of the surgical sponges and the surgical instruments [1].

This issue will make an economic and legal burden on the health care providers and health institutions even if it causes no morbidity or minor adverse effects [1].

Surgical sponges are the commonest retained foreign bodies, while other types like surgical needles and instruments are not very common [1].

The clinical presentation varies according to the site of the surgery, the surgical procedure, and the indications for the surgery. Intra-abdominal retained surgical sponges induce an exudative tissue response which may be infected later on, patients have abdominal pain, fever, anorexia, nausea, vomiting, and there may be abdominal distension and intestinal obstruction. In addition to various clinical presentations, this causes a major emotional distress to the patient, the family, and the health care providers. [1,3].

Imaging is the main diagnostic tool. CT-scan is the imaging modality of choice, plain abdominal X-ray films may show the radio-opaque marker, although the false negative rate may reach 25 % [1].

The work of this report case has been reported in line with the SCARE 2018 criteria [4].

## 2 Patient information

A 40-year-old lady presented with abdominal pain, diarrhea and bilious vomiting for 3 days. The pain was colicky in nature and radiated to the flanks, it was relieved by vomiting. The patients had history of cesarean section which was performed before 4 months, the cesarean section was difficult because of the transverse lie of the baby.

### 2.1 Clinical findings

The patient was admitted to the medical department for one day, then referred to the surgical department. During examination the pulse rate was 90 beats/minute, the blood pressure was 100/60 mmHg, and the temperature was 37.5 °C.

During general examination; the patient was pale with no jaundice. Abdominal examination showed tenderness in the lower abdomen with muscle guarding, with no distension.

### 2.2 Diagnostic assessment

The hemoglobin level was 8.6 g/L, the WBC count were 8700 c/mm, the renal function test and the electrolytes were normal. Urinalysis showed evidence of one plus pus cells per high power field, with no RBC or crystals in the examined sample.

**Fig. 1:**
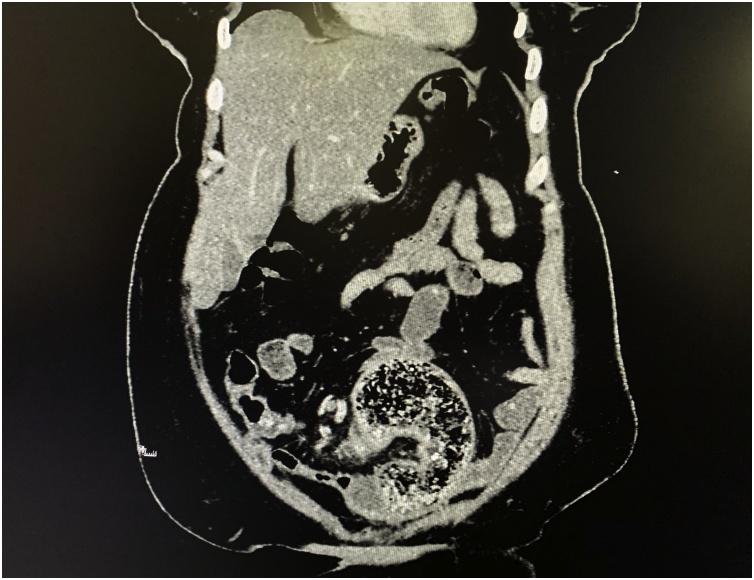
CT-scan of the abdomen showing an evidence of thickened wall of the sigmoid colon with intramural air, the proximal small bowel show dilatation.

Abdominal ultrasound showed no abnormal findings apart from mild hydronephrosis in the left side. CT-scan of the abdomen showed thickening of the wall of the sigmoid colon with evidence of intramural air and dilated small bowel loops. Other organs showed no abnormalities. Although the surgical sponge contained a radio-opaque line, but it was not evident in CT-scan, possibly because it was mixed with hard fecal material and air (Fig. 1).

**Fig. 2:**
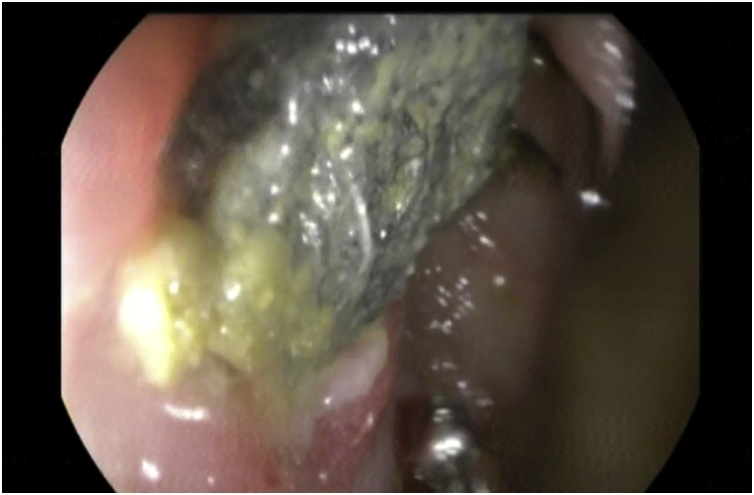
A colonoscopic view showing the surgical sponge causing erosion and ulceration in the wall of the sigmoid colon.

During colonoscopy there was an evidence of a surgical sponge causing transmural erosion and ulceration of the wall of the sigmoid colon (Fig. 2).

The past medical history was negative for chronic illnesses and patient had previous history of appendicectomy and 2 cesarean sections.

### 2.3 Therapeutic intervention

**Fig. 3:**
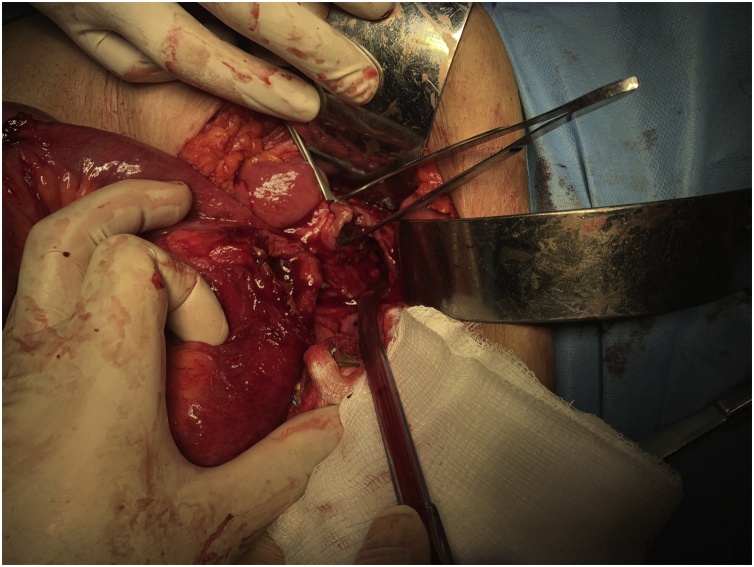
An intraoperative picture showing the fistula between the ileum and the sigmoid colon.

**Fig. 4:**
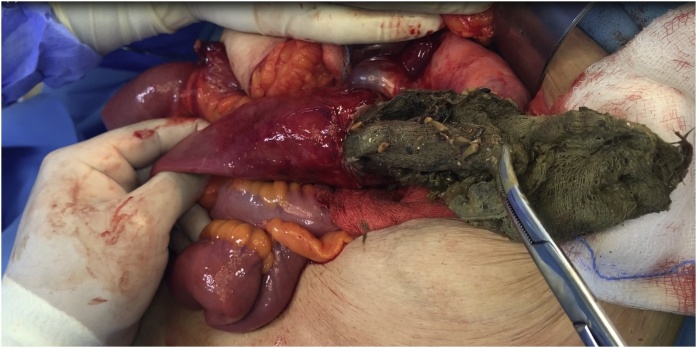
An intraoperative picture showing the retained surgical sponge being extracted from the lumen of the bowel.

**Fig. 5:**
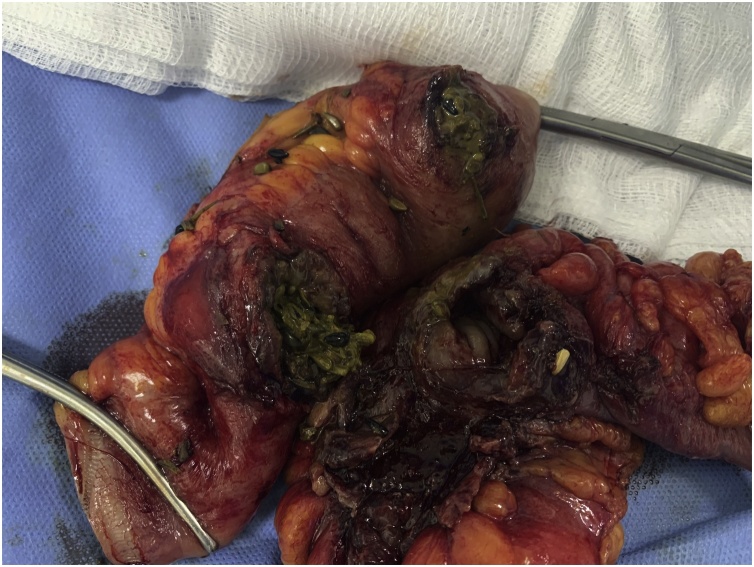
An intraoperative picture showing the resected segment of the ileum and the sigmoid colon.

**Fig. 6:**
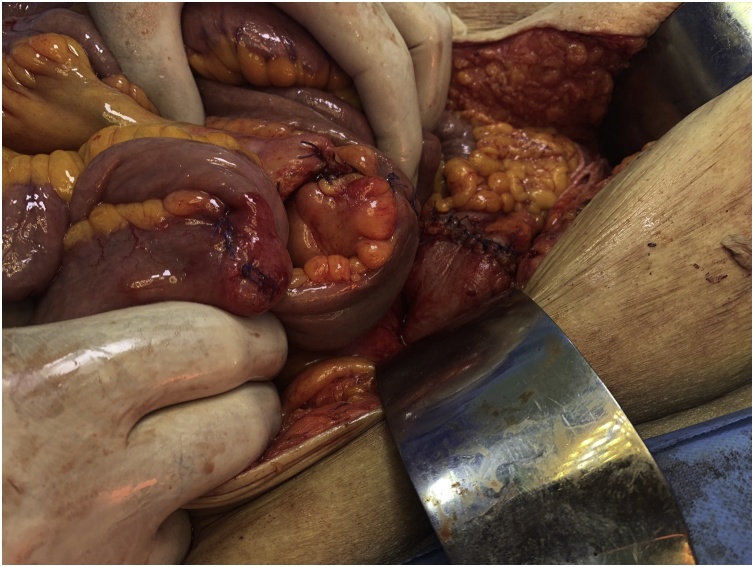
An intraoperative picture showing the anastomosis sites of the ileum and the sigmoid colon.

Exploratory laparotomy was performed. During surgery there was an evidence of a retained surgical sponge in the pelvic cavity causing erosions and fistula between the ileum and the sigmoid colon. Resection of the involved parts of the ileum and the sigmoid colon was done with end-end anastomosis (Fig. 3, Fig. 4, Fig. 5, Fig. 6).

We contacted the obstetric team about our finding and they reported it in the hospital records.

### 2.4 Follow-up and outcomes

The patient was admitted for one week after surgery, with improvement of the general condition and returning of the normal bowel function and was discharged home after that. At the 10th postoperative day, she developed complete abdominal dehiscence and she was readmitted to the emergency department, resuscitation was done and an emergency operation was performed. The abdomen was closed with tension sutures. The patient was admitted for 5 days and was then discharged home later with good medical condition.

The tension sutures were removed after 3 weeks and the patient was followed for 1 month after that with no further complications.

## 3 Discussion

The causes of retained surgical sponges are studied by many authors, the most common risk factors include emergency surgeries, surgery performed for obese patients, unexplained intraoperative change in the surgical procedure, incorrect count of the sponges before closure, and when multiple procedures performed for the same patient [1].

Sharp instrument cause more serious sequelae and have earlier presentation than other types [1].

The retained surgical sponges may remain asymptomatic for many years, some are discovered accidentally, or during performing a surgery for something unrelated, alternatively they may have serious presentations like major sepsis, intestinal obstruction, fistulation, and even death have been reported [3].

There are many methods which are adopted to decrease the rate of the retained surgical sponges and instruments, such as counting, postoperative high resolution X-ray screening, radiofrequency identification, electronic means, and the use of barcoded or data matrix coded surgical sponges [1,3,5,6].

The current minimum required recommendations for the operating room nurse is 3 separate counts for all potential surgical sponges and instruments, one before the surgery, one during the procedure, and one once the incision is closed, in case of any miscount, intraoperative X-ray of the body region involved is required. Reliance on a single technique is not recommended by many authors and some centers perform routine postoperative X-ray [3,7].

The exact number of the cases is underreported by most of the medical institutions mainly due to privacy issues and legal concerns. More than half of the preventable events listed as “never events” occur during surgery, other may occurs in other medical departments such as sending home the wrong newborn with the wrong family. Some medical institutions are obligated by law to report all these events [3].

The surgical team is responsible for preventing this event by careful inspection of the surgical site using all the available methods and technology. Technology increases the safety but doesn’t accurately prevent the accidents. All causative human and technical factors must be addressed carefully [3,5].

### 3.1 Patient’s perspective

After surgery I am afraid of recurrence of the cyst, I should keep regular checks with my doctor.

## Sources of funding

None.

## Ethical approval

Ethical approval has been exempted by my institution for reporting this case.

## Consent

Written informed consent was obtained from the patient for publication of this case report and accompanying images.

## Author contribution

The concept of reporting the case and data recording was done by Dr Asaad Sahreef Omar and Dr Ayad Ahmad Mohammed.

Drafting the work and final approval of the work to be published was done by Dr Ayad Ahmad Mohammed.

## Registration of research studies

This work is case report and there is no need of registration.

## Guarantor

Dr Ayad Ahmad Mohammed is guarantor for the work.

## Provenance and peer review

Not commissioned, externally peer-reviewed.

## Declaration of Competing Interest

The author has no conflicts of interest to declare.
